# *Trichormus variabilis* (Cyanobacteria) Biomass: From the Nutraceutical Products to Novel EPS-Cell/Protein Carrier Systems

**DOI:** 10.3390/md16090298

**Published:** 2018-08-27

**Authors:** Erika Bellini, Matteo Ciocci, Saverio Savio, Simonetta Antonaroli, Dror Seliktar, Sonia Melino, Roberta Congestri

**Affiliations:** 1 Laboratory of Biology of Algae, Department of Biology, University of Rome Tor Vergata, 00133 Rome, Italy; erikabellini1990@gmail.com (E.B.); saverio.savio@gmail.com (S.S.); 2 Department of Chemical Science and Technologies, University of Rome Tor Vergata, 00133 Rome, Italy; ciocci.matteo@gmail.com (M.C.); simonetta.antonaroli@uniroma2.it (S.A.); 3 Department of Biomedical Engineering, Technion Israel Institute of Technology, 3200003 Haifa, Israel; dror@bm.technion.ac.il; 4 CIMER, Center of Regenerative Medicine, University of Rome Tor Vergata, via Montpelier 1, 00133 Rome, Italy

**Keywords:** Extracellular Polymeric Substances, hydrogel, mesenchymal stem cells, biomaterials, enzyme, omega 3, PUFA, *Trichormus variabilis*, Cyanobacteria

## Abstract

A native strain of the heterocytous cyanobacterium *Trichormus variabilis* VRUC 168 was mass cultivated in a low-cost photobioreactor for a combined production of Polyunsaturated Fatty Acids (PUFA) and Exopolymeric Substances (EPS) from the same cyanobacterial biomass. A sequential extraction protocol was optimized leading to high yields of Released EPS (REPS) and PUFA, useful for nutraceutical products and biomaterials. REPS were extracted and characterized by chemical staining, Reversed Phase-High-Performance Liquid Chromatography (RP-HPLC), Fourier Transform Infrared Spectroscopy (FT-IR) and other spectroscopic techniques. Due to their gelation property, REPS were used to produce a photo-polymerizable hybrid hydrogel (REPS-Hy) with addition of polyethylene glycol diacrylated (PEGDa). REPS-Hy was stable over time and resistant to dehydration and spontaneous hydrolysis. The rheological and functional properties of REPS-Hy were studied. The enzyme carrier ability of REPS-Hy was assessed using the detoxification enzyme thiosulfate:cyanide sulfur transferase (TST), suggesting the possibility to use REPS-Hy as an enzymatic hydrogel system. Finally, REPS-Hy was used as a scaffold for culturing human mesenchymal stem cells (hMSCs). The cell seeding onto the REPS-Hy and the cell embedding into 3D-REPS-Hy demonstrated a scaffolding property of REPS-Hy with non-cytotoxic effect, suggesting potential applications of cyanobacteria REPS for producing enzyme- and cell-carrier systems.

## 1. Introduction

Cyanobacteria are known as the most abundant phototrophic organisms in the Ocean. They are versatile and successfully colonize a wide range of aquatic and terrestrial habitats also thriving in strongly fluctuating environments, including the most extreme habitats on Earth [1]. Cyanobacterial diversity is enormous and represents a source of biotechnologically important organisms for new products and applications [2,3]. Commercial exploitation of cyanobacteria relies on intensive cultivation of biomass for the production of high-value compounds useful not only in nutraceutics, therapeutics and cosmetics but also for the production of advanced bio-material [4,5,6,7,8,9,10]. In this context, we focused on a strain of *Trichormus variabilis* (Kützing ex Bornet & Flahault) Komárek & Anagnostidis, isolated from sediment biofilms of a dystrophic coastal lagoon [11]. *T. variabilis* VRUC168 was selected based on prior studies that showed ease of growth in a range of photobioreactors (PBRs), even in suspension, self-flocculation, and interesting productivity of nutraceutical products, such as Polyunsaturated fatty acids (PUFA) and Exopolymeric Substances (EPS) [12,13]. 

EPS may constitute up to 60% of the dry biomass (as in the case of *Nostoc commune* and *Trichocoleus sociatus*) [14,15] and can be tightly bound (cell-attached or capsular), loosely adhere (slime type) to cells or exist as free dissolved matter called Released EPS (REPS). We focused on REPS that are usually recovered from the liquid growth media of cyanobacterial cultures with a green, environmentally safe process without using chemicals [16,17].

In the past few years, several studies have demonstrated a high potential application of cyanobacterial EPS that consist of various organic substances: mainly extracellular polysaccharides, uronic acids, proteins, nucleic acids and lipids [16]. Generally, they are characterized by a high complexity in terms of monosaccharidic composition. EPS can contain up to 15 sugar moieties, organized in complex repeating units and are often characterized by a high molecular weight, of up to 1–2 MDa [18]. The presence of hydrophilic moieties on one side (sulfated sugars, uronic acids and ketal-linked pyruvyl groups, among others), and hydrophobic on the other (acetyl groups, dehoxysugars and peptides) confers an amphiphilic character to the macromolecules and hence provides greater plasticity in organisms’ response to surrounding environment [19]. While sulfate groups and uronic acids contribute to the anionic nature of the EPS, conferring a negative charge and a “sticky” behavior to the overall macromolecule [20,21,22], hydrophobic compounds are responsible for their emulsifying and rheological properties [23,24]. Due to these features, EPS are also used for the production of emulsifiers, viscosifiers, soil conditioners, biosorbants and bioflocculants [18]. Cyanobacterial EPS can play diverse roles in vivo; they form a three-dimensional network holding cells together and mediating their attachment to exposed surfaces [12,25,26]. A recent application of EPS-rich cyanobacteria, related to the physiological role of the EPS, is their use as nutrient supplements and physical soil amendments for the recovery of eroded soils [27,28,29]. EPS hydration and rheological properties are important to prevent cell desiccation and to confer pseudoplastic behavior of the extra-cellular environment [18,19,30]. These polymers are also involved in other relevant physiological roles from the UV protection and antibiotic resistance, to the mechanical strength and exo-enzymatic degradation activity [18,31,32], which could be of interest for biomedical applications. Although these substances are widely studied, in fact, the potential applications of the EPS and REPS are not completely understood. Due to their high content of polysaccharides, these polymers are highly promising materials for applications in biomedicine and tissue engineering [33,34]. Indeed, the ability of natural polysaccharides to form hydrogels, in which three-dimensional (3D) cross-linked network structures retain a large amount of water, makes them very useful for the production of drug- or cell-carrier systems and scaffolds. In particular, therapeutic molecules or macromolecules (proteins and nucleic acids) can be entrapped into the inner structure of these hydrogels or adsorbed onto their external surface, to facilitate better targeting to organs and tissues. The embedding of active molecules into polysaccharide gels usually increases their availability, also permitting for the drug administration at lower doses and, consequently, the reduction of the toxicity for the patient [35]. Moreover, hydrogels have become important as cell-carrier systems [36,37] for the transplantation of cells in the therapy of a variety of diseases (e.g., liver failure and diabetes) [38]. In our study, a novel photo-polymerizable REPS hydrogel was produced, combining the properties of natural REPS from *T. variabilis* with those of the synthetic polyethylene-glycol diacrylated (PEGDa). Here, we investigated the chemico-physical and mechanical properties of this hybrid hydrogel and its potential applications in production of detoxification enzyme- and stem cell-carrier systems. Therefore, the feasibility of an integrated approach that combines the cyanobacterial biomass production with the extraction of unsaturated FA and the fabrication of EPS hydrogels for enzyme- and cell-carrier systems are demonstrated. 

## 2. Results and Discussion

### 2.1. T. variabilis Growth and Biomass Yields in the PBR

**Figure 1:**
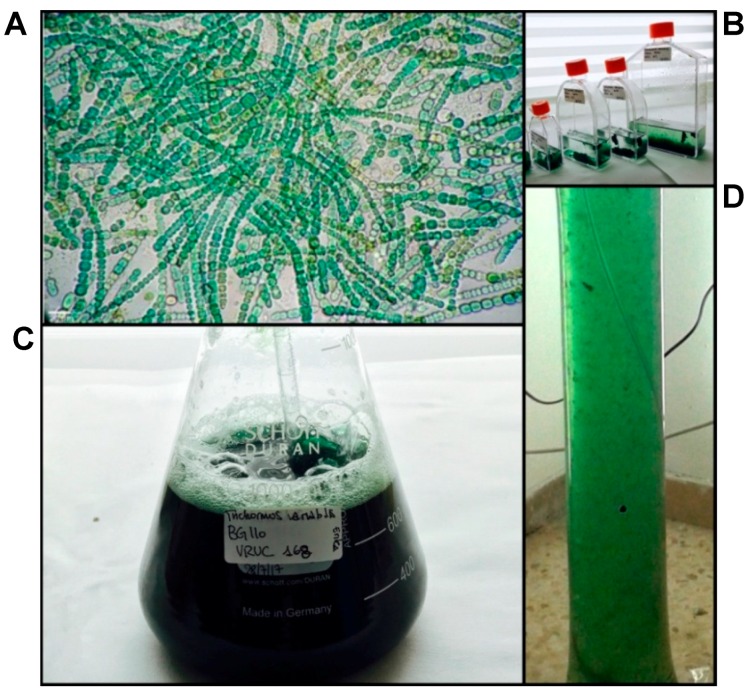
Biomass production. Light micrograph of *T. variabilis* trichomes in: culture (**A**); bench-scale growth system used (**B**,**C**); and pilot-scale growth system used (**D**).

**Figure 2:**
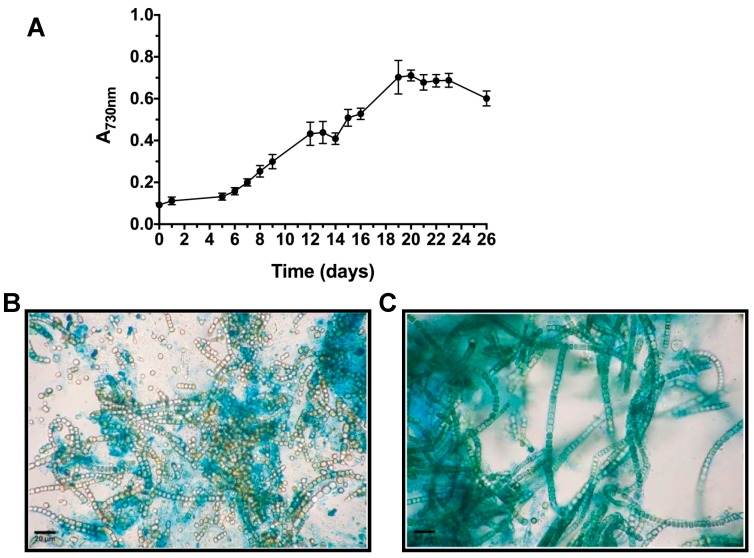
Growth curve recorded in polyethylene bag growth experiment (**A**). Light micrographs of cultures after Alcian Blue staining at pH 0.5 (**B**) and 2.5 (**C**) for sulfated and carboxylic EPS residues, respectively. Sulfated EPS were diffluent and less abundant (**B**), while carboxylic groups were observed in more bound matrix material (**C**).

*T. variabilis* VRUC168 showed the ability to grow intensively and in suspension in the low-cost, 10 L polyethylene vertical bags used in this study (Figure 1). These growth systems allowed optimization of the space occupied by the culture and of the illumination provided. Although biofilm growth systems have recently proved more productive [14,39] to investigate potential employment of REPS as advanced bioactive material, the selected PBR configuration and material appeared satisfactory. Indeed, in our experiment, nutrient provision occurred only at the start of the PBR growth to test further reduction in biomass costs and open the path to a scale-up of *T. variabilis* biomass production for extraction of valued products [7,9]. Growth curves showed that the exponential growth reached its maximum after 20 days of culture (Figure 2A), when the maximum production of 0.787 ± 0.010 gDW L^−1^ was also measured.

Data obtained for the filamentous, non-heterocytous, cyanobacterium *Arthrospira* sp. grown in a 5 L reactor with air mixing show lower biomass production (0.67 ± 0.03 g L^−1^), but higher rates, reaching exponential phase after only four days [40]. Studies conducted on mass cultivation of other filamentous forms report lower biomass and daily productivity values, as in the case of *Limnothrix* sp., grown in a 3.5 L PBR system (0.02 gDW L^−1^ d^−1^; 0.29 gDW L^−1^) [41] and *Oscillatoria* sp. in suspension, with 0.26 gDW L^−1^ produced after 20 days [42]. Our results show the ability of *T. variabilis* to grow in a simple intensive growth system and to produce rapidly a sufficient amount of biomass to be exploited for biotechnological applications.

### 2.2. FA Extraction and Characterization

Among several metabolites produced from cyanobacteria, PUFA have gained much consideration due to their nutritional importance. Particularly, single FA are valued in food and pharmaceutical production due to their antioxidant, anti-inflammatory and anti-microbial activities [43,44]. With the aim of combining EPS production with that of FA from the same biomass, Fatty Acid Methyl Esters (FAME) were extracted and characterized as co-products. The total FAME content rather than its pattern is known to be dependent on the species and growth conditions, and is often considered as the most important factor for industrial applications [45,46]. In this study, no culture stress to induce lipid production was applied and 63.44 ± 0.46 mg/gDW (6.34% *w*/*w*) of FAME were obtained from *T. variabilis* biomass. Our results were comparable to those obtained by Gayathri and colleagues [47] for the same species (reported as its synonym *Anabaena variabilis*) while were higher than those reported by the same authors for other heterocytous cyanobacteria, *Nostoc commune* (about 1.49% *w*/*w*) and *Nostoc muscorum* (about 5% *w*/*w*), grown without stress. The FAME composition of *T. variabilis* is shown in Table 1, with higher amounts of PUFA (57.45 ± 4.77%) obtained as compared to both monounsaturated FA (MUFA) (18.29 ± 0.02%) and saturated FA (SAFA) (24.25 ± 4.76%). The MUFA and PUFA, here produced in high yields were hexadecanoic acid (16:1) (15.25 ± 1.34%), octadecanoic acid (18:1; 3.05 ± 1.36%), octadecadienoic acid (18:2; 24.46 ± 1.91%), and octadecatrienoic acid (18:3, 27.71 ± 2.33%), which are also relevant FA for industrial production [48,49]. These yields were higher than those obtained without stress in *Anabaena cylindrica*, *Aphanizomenon gracile* and *Nostoc muscorum* [50]. Moreover, linoleic acid (C18:2 (n-6)) and α-linolenic acid (C18:3 (n-6)) were the most abundant FA in the *T. variabilis* FAME profile. From the biological activity point of view, ω-3 and ω-6 FA are essential nutritional components that display important functions in the human metabolism [51], such as in the regulation of oxygen and electron transport and membrane fluidity [52,53]. Therefore, they can be effective in cardiac protection [54,55] and cancer prevention [56,57,58,59], type 2 diabetes, inflammation and obesity [53,60,61]. These results suggest the possibility to use *T. variabilis* biomass as a low-cost source of PUFA for nutraceutical applications and to integrate the production of REPS with that of PUFA. This allows reducing operational costs and making the exploitation of the studied cyanobacterial strain economically more advantageous.

### 2.3. EPS Extraction and REPS Characterization

The ability to synthesize relevant amounts of highly heterogeneous, hydrated and charged EPS plays critical roles in cyanobacterial cellular cohesion, protection and metabolic integrity. In response to water availability, EPS undergo striking changes in their rheological properties [62]. A first insight into their variable composition can be obtained by microscopic observations of cyanobacterial biomass after specific staining. Cytochemical characterization was performed using Alcian Blue (AB) at two pH values (Figure 2B,C). AB staining at 2.5 pH revealed the presence of carboxylic groups in the EPS material adherent to *T. variabilis* cell surface (bound EPS), while AB at 0.5 pH reacted with sulfated residues evidencing a lower amount in the released material, confirming what was previously observed for the same strain in our laboratory [12]. 

**Figure 3:**
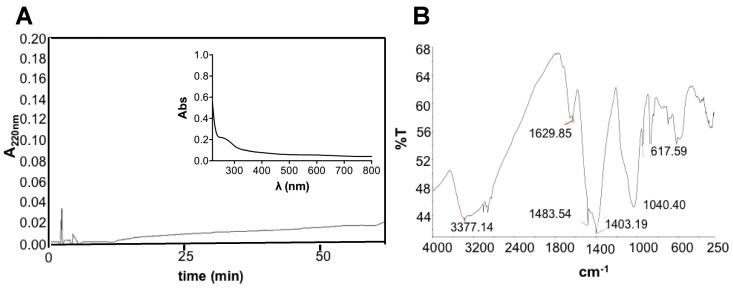
Characterization of REPS: (**A**) RP-HPLC chromatogram of REPS solution (10 mg/mL) using C_18_ column at 0.8 mL/min flow rate and the following gradient: 0–5 min, 0%; 5–50 min, 60%; 50–55 min, 60%; 55–60 min, 90%; and 60–65 min, 90% of solvent B (80% *v*/*v* CH_3_CN and 0.1% *v*/*v* TFA). Inset: UV-vis absorption spectrum of REPS solution. (**B**) FT-IR spectrum of REPS showing signals within 4000 to 250 cm^−1^; the measurements were consistent among three replicates.

The obtained REPS after 20 days of growth were 465 mg/gDW and the plot of REPS production over growth is shown in Figure S1. Previous data on the same strain grown at bench-scale, without air mixing, showed lower REPS content, of about 86.7 mg/gDW [12]. Therefore, our data, in agreement with Pereira and colleagues [18], would evidence that the culture turbulence, due to the aeration, may facilitate the release of EPS from the cell surface and stimulate their synthesis. The REPS produced by *T. variabilis* in this study were about 46.5% of the dried biomass, a value comparable to that recently obtained from *Trichocoleus sociatus* (60%) grown in an aerosol-based emerse photobioreactor (ePBR) to simulate this terrestrial cyanobacterium natural environment [14]. It has to be noted that that the proportion and composition of released and bound EPS can show high variability depending on external environmental/cultivation factors and on the strain itself. Indeed, a combination of drought and salt stress was successfully used to increase EPS production in *Trichocoleus sociatus* [14,63]. Previous data on the studied strain cultivated at smaller scale without any aeration showed a complex monosaccharide composition of its exopolysaccharides that were composed of ten different residues whose glucose and xylose were the most abundant and uronic acids, such as galacturonic acid and glucuronic acid, that contributed to their anionic nature and sticky character [12]. Figure 3A shows the RP-HPLC and the spectrophotometric data of the REPS solution demonstrating the absence of both hydrophobic compounds and chromophores. The low peptide or protein content was also confirmed by bicinchoninic acid (BCA) assay resulting in a protein concentration of 0.39 ± 0.02 mg/mL, corresponding to 3.6% *w*/*w* of REPS dry weight. Sugar content was assessed using phenol method, showing 10.2% *w*/*w* of REPS dry weight. Infrared spectroscopic analysis of the REPS powder, obtained after freeze-drying, was also performed (Figure 3B).

The FT-IR spectrum confirmed the presence of polysaccharides showing the presence of specific absorption bands. The observed bands were characteristic of carbohydrates (1403 cm^−1^ and 1040 cm^−1^) and polymeric substances (3377 cm^−1^) with –OH, –COOH, phenolic and –CH_2_ groups [64,65,66,67]. In particular, the signal around 1040 cm^−1^ suggested the presence of carbohydrates with sulfur functional groups confirming what was observed after cytochemical staining in light microscopy. A peak around 3377 cm^−1^ was attributed to stretching vibration of hydroxyl groups, characteristic of –OH groups into polymeric substances. Furthermore, bands at 2915–2935 cm^−1^ were due to asymmetrical C–H stretching vibration of aliphatic CH_2_-group [64,65]. Moreover, a band at 1629 cm^−1^ of the amide I region was observed, probably suggesting the presence of proteins [66]. The presence of several peaks at wavelengths lower than 1000 cm^−1^ may be due to several visible bands attributed to phenolic groups, phosphate functional groups and/or to the occurrence of possible linkages between monosaccharide units [68]. Furthermore, the presence of carboxylic groups suggests, when combined with the other observed bands, the presence of uronic acids (especially with sugar-characteristic bands) and of humic substances (–CH_2_ and phenolic groups).

### 2.4. REPS-Hy Synthesis and Characterization

**Figure 4:**
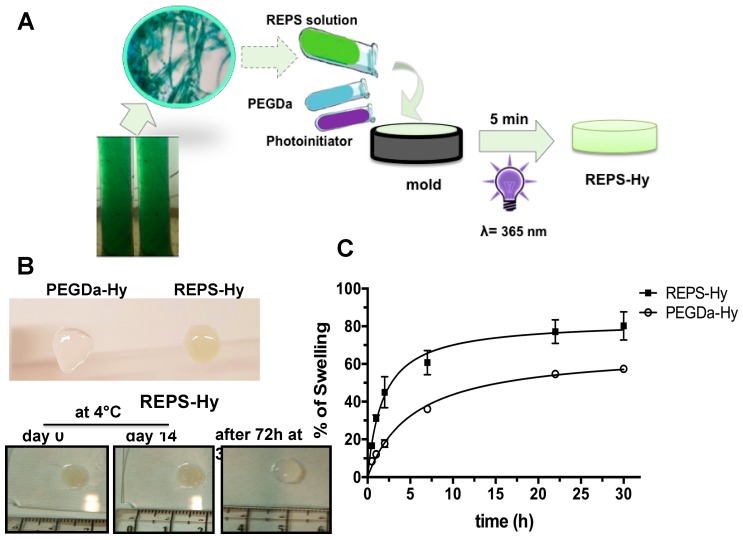
Synthesis and characterization of the REPS-Hy: (**A**) Schematic representation of REPS-Hy production: the gelling of the solution of 8.83 mg/mL of REPS with 3% of PEGDa (6 kDa) (*w*/*v*), and 0.1% of Irgacure^®^2959 (*w*/*v*) was obtained after 5 min of UV light (365 nm) exposition. (**B**) Resistance of REPS-Hy to dehydration and spontaneous hydrolysis. Digital macro-photographs of: PEGDa-Hy with 2% of PEGDa without REPS and REPS-Hy with 2% of PEGDa (*w*/*v*) (**top**); REPS-Hy with 3% of PEGDa-Hy (*w*/*v*) at Day 0 and Day 14 after storage at 4 °C, and after 72 h of incubation at 37 °C in PBS (**bottom**). (**C**) Swelling rate curves of REPS-Hy and PEGDa-Hy in PBS up to the equilibrium swelling (30 h). The R^2^ of the hyperbolic fits of the swelling trend of REPS-Hy and PEGDa-Hy are 0.9284 and 0.9912, respectively.

Generally, many polysaccharides are characterized by gelling property that was investigated by preparing several solutions at different concentrations of REPS and pH values. Although a gelation tendency was observed at 68.67 mg/mL in H_2_O, the REPS gel was not stable. Therefore, hybrid hydrogels made of 8.0 mg/mL of REPS solution and 2% or 3% (*w*/*v*) of PEGDa (6 kDa) were prepared by UV photo-polymerization, according to the schematic representation shown in Figure 4A. The presence of REPS was relevant for the gelation process, as shown in Figure 4B, wherein an unstable hydrogel obtained after photo-polymerization of a solution with 2% (*w*/*v*) of PEGDa without REPS (Figure 4B, top) is compared to a stable REPS-Hy with 3% of PEGDa. The stability of the REPS-Hy in PBS was assessed both over two weeks at 4 °C and after 72 h at 37 °C (Figure 4B, bottom); neither condition exhibited spontaneous hydrolysis.

Moreover, REPS-Hy was resistant to dehydration and morphological changes were not detectable also after long incubation times (Figure 4B). The REPS-Hy degree of swelling was analyzed over time (Figure 4C) and compared to that of PEGDa-Hy, which was obtained using 3% of PEGDa (6 kDa). The percentage of water-uptake (%WU) at the “equilibrium swelling” (% S_eq_), was corresponding to 80.20 ± 7.5% WU for REPS-Hy in PBS after 22 h of hydration. Moreover, the swelling of REPS-Hy was about 22.8 ± 6.5% more than that observed for PEGDa-Hy. These results agree with the natural hydration properties of the EPS from cyanobacteria and such properties are crucial for the survival of these organisms. Important physiological properties, in fact, have been attributed to EPS including the physical barrier to the environment and the desiccation tolerance, as well as the subsequent rehydration [62,69]. These properties highly stabilize cells during long-term storage in the air-dried state [62]. The rheological properties of EPS are strictly related to their 3D supramolecular structure, which changes in response to environmental variables regulating the mass transfer and other biophysical properties that modulate cell activity. Therefore, the intrinsic EPS properties preserved in our REPS-Hy could be important in biomedical applications as well as in the emerging field of 3D-bioprinting. 

**Figure 5:**
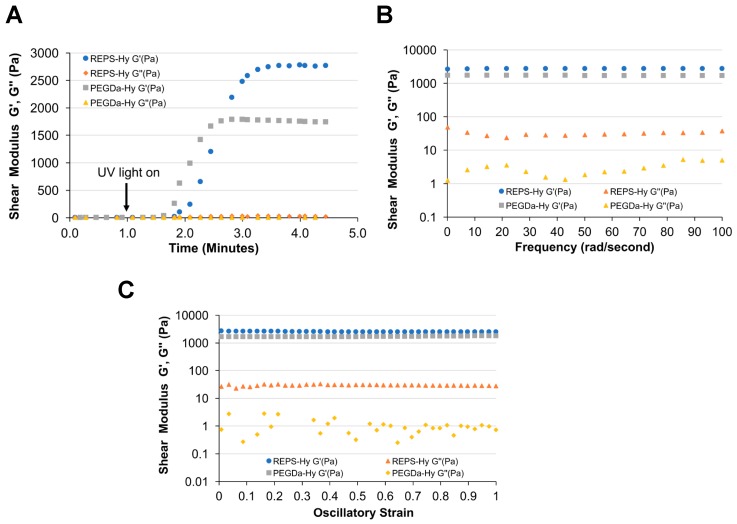
REPS improve hydrogel mechanical properties. Rheological measurement of REPS-Hy and PEGDa-Hy as evaluated by: time-sweep tests (**A**); frequency-sweep tests (**B**); and strain-sweep tests (**C**). The shear storage modulus (G′) and shear loss modulus (G′′) are shown for both hydrogels. (**A**) The time sweep data reveal an increase in G′ upon the light-activated free-radical polymerization reaction of the REPS-Hy and PEGDa-Hy liquid precursors. The plateau G’ of the REPS-Hy was 55% higher as compared to the plateau G′ of the PEGDa-Hy, indicating that the REPS improves the elastic mechanical properties of the hydrogels. Following the chemical cross-linking of the hydrogels, the frequency-sweep (**B**) and strain-sweep (**C**) data confirmed a linear relationship between the shear modulus in the range of the applied frequency and strain.

Rheological analysis of REPS-Hy and PEGDa-Hy (without REPS) reveals differences in the shear storage modulus (G’) that are likely associated with additional crosslinking in the REPS-Hy (Figure 5). The plateau storage moduli of the REPS-Hy and PEGDa-Hy were 2778 Pa and 1785 Pa, respectively (Figure 5A). The average shear loss modulus of the REPS-Hy was also higher than that of the PEGDa-Hy. This order of magnitude increase in shear loss modulus is likely attributed to higher viscosity associated with high molecular weight REPS macromolecules. The frequency and strain sweep rheological analysis confirms that the REPS-Hy and PEGDa-Hy both display similar viscoelastic behavior, notably that both show a uniform stress response to alternations in strain or frequency of oscillatory deformation (Figure 5B,C). Therefore, it is assumed that the REPS do not alter the polymer network structure beyond those attributes associated with the additional cross-linking and increased macromolecular chain length of the REPS.

### 2.5. REPS-Hy as Enzyme-Carrier System

**Figure 6:**
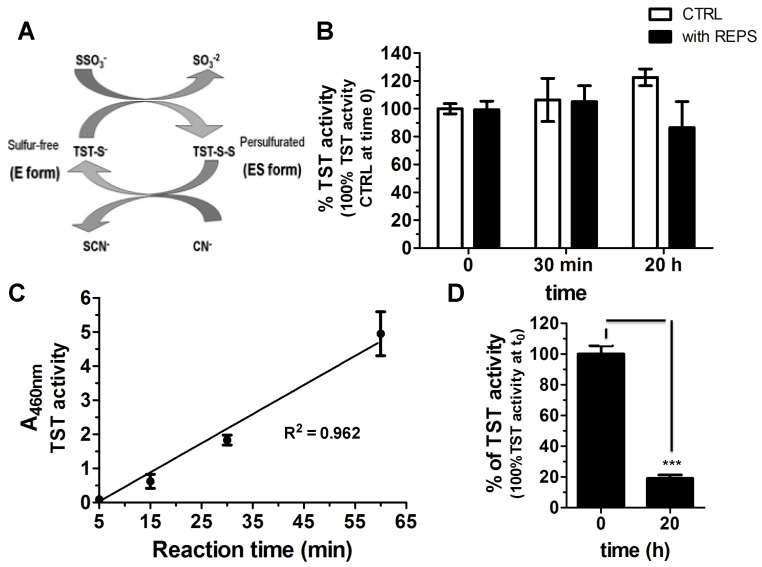
REPS-Hy as detoxification enzyme-encapsulating hydrogel: (**A**) Scheme of the catalytic cycle of the TST enzyme. (**B**) Percentage of TST activity over time of 3.12 μM of TST in presence of 50 μL of PBS (white) or of REPS solution (8 mg/mL of REPS and 8 mM of CaCl_2_ in PBS, pH 7.4) (black) (100% is the activity in PBS at time 0). The Sörbo assay was performed at 23 °C. (**C**) TST activity of TSTREPS-Hy at different incubation times (5, 15, 30 and 60 min) at 37 °C. The reaction was stopped after incubation and the absorbance of the solutions measured after dilution. The line equation is *y* = 0.07611x and R^2^ is 0.9621. (**D**) TST activity of TSTREPS-Hy at time 0 and after 20 h at room temperature (23 °C) in 200 µL of 50 mM Tris-HCl, pH 8.0, buffer (100% is the TSTREPS-Hy activity at time 0). *** *p* < 0.001, *n* = 3 or 5.

REPS-Hy was studied as a potential enzyme-carrier system and the detoxification enzyme thiosulfate:cyanide sulfurtranferase (TST) was used as enzymatic model. The recombinant TST from *Azotobacter vinelandii* used herein is characterized by the presence of only one Cys residue, which is also the catalytic residue present in the active site. Figure 6A shows the scheme of its enzymatic activity. First, the TST activity was assessed in the presence and in the absence of REPS solution (8.0 mg/mL REPS, 8 mM CaCl_2_ in PBS) at room temperature at different times of incubation (0 min, 30 min and 20 h) (Figure 6B). The REPS solution did not significantly affect the TST activity, even after many hours of incubation (Figure 6B). These results suggested us the possibility to embed the TST into REPS-Hy. The TST enzyme solution was mixed with REPS-Hy precursor solution and, after photo-polymerization, the TST activity was evaluated using the Sörbo assay at different incubation times in the presence of the substrates (1, 5, 15, 30 and 60 min) at 37 °C (Figure 6C). 

TSTREPS-Hy showed enzymatic activity (Figure 6D), although a statistically significant decrease of the enzymatic activity of the embedded TST in the gel with respect to the TST in solution was observed. This was probably due to both the photo-polymerization process and to the diffusion rates of the substrates and the final product. 

A linear increase of the TST activity of the TSTREPS-Hy in a time-dependent manner was observed, likely owing to the diffusion phenomena in the gel (Figure 6C). Accordingly, the selected time to perform the activity assay of TSTREPS-Hy was 30 min, which was a good compromise to have a detectable TST activity, minimizing the effect due to diffusion of the substrates and products in and out the gel and at least avoid the dilution for the measure of the absorbance. The enzymatic activity of TSTREPS-Hy was assessed over time (Figure 6D), and after 20 h of incubation into 200 μL of 50 mM Tris-HCl buffer, pH 8.0, at 37 °C only the 20% of the TST activity was recovered. The decrease of the enzymatic activity could be due to the release of the enzyme from the gel considering that the REPS solution did not induce a statistically significant inhibition of the enzymatic activity. The RP-HPLC analysis of the soluble fraction was performed, but unfortunately the TST release was not detectable for the overlapping of the retention times of TST peak with PEGDa-derivative molecules that were released from the gel (Figure S2). These preliminary results suggest the possibility to use the REPS-Hy as TST carrier system for the cyanide detoxification. A rapidly growing class of modern therapeutics is constituted by proteins and peptides, which often show a better efficiency than small drugs for the therapy and diagnosis of serious and deadly diseases. However, their administration is often difficult due to the loss of the native structure and rapid cleavage in the liver or other body tissues. Their instability could be diminished by biocompatible and biodegradable hydrogels useful as carriers able to improve their bioavailability and to provide other routes of administration [70]. Several proteins including insulin, growth hormones, and interferons at other proteins have been already encapsulated in microbial exopolysaccharide hydrogel particles [71,72,73,74] and the improving their administration has been demonstrated. In this context, the results herein proved activity of the detoxification enzyme either in the presence of REPS solution from *T. variabilis* or into REPS-Hy. They suggest the possibility to use EPS for the production of hydrogel carrier systems of enzymes characterized by the presence of cysteine at the catalytic site, although other experiments, using different enzymes, are necessary to support this hypothesis. Moreover, the enzyme-REPS-Hy could represent a good opportunity to combine the EPS natural properties, such as antibacterial, anti-oxidative and anti-inflammatory properties [75], with a specific enzymatic activity. 

### 2.6. REPSHy as Stem Cell Carrier for Tissue Engineering

**Figure 7:**
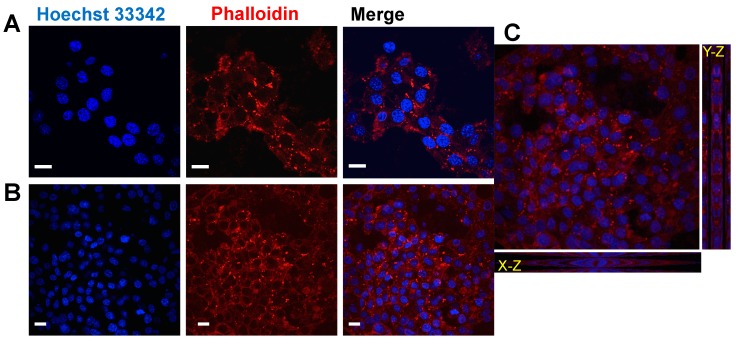
hMSCs cultures using REPS-Hy as scaffold. Confocal laser scanning micrographs of hMSCs cultured for two weeks on REPS-Hy: (**A**) at 60× magnificantion; (**B**) at 40× magnification; and (**C**) micrographs with Y–Z and X–Z projections. F-actin was stained with Alexa-fluor 568 phalloidin-conjugate (in red) and the nuclei were stained using Hoechst 33342 (in blue). Scale bars = 10 μm.

The biocompatibility of REPS-Hy was also evaluated by analysis of the cell adhesion of human mesenchymal stem cells (hMSCs) seeded onto REPS-Hy and cultured for two weeks in the presence of DMEM medium. Figure 7 shows the confocal laser scanning micrographs of hMSCs after two weeks of growing on REPS-Hy. 

**Figure 8:**
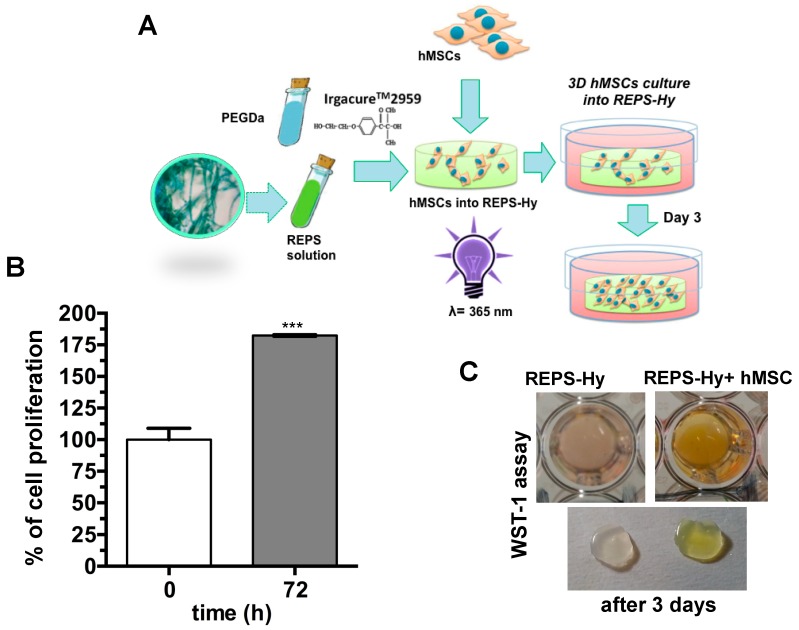
REPS-Hy as stem cell-carrier system: (**A**) Schematic representation of the production of REPS-Hy scaffolds for 3D hMSC cultures. (**B**) Cell viability of hMSCs embedded into the REPS-Hy, immediately after photo-polymerization (time 0) and after 72 h of 3D cell culture (100% is the cell viability at time 0). (**C**) Digital macro-photographs of REPS-Hy with and without embedded hMSCs after colorimetric WST-1 cell viability assay. *** *p* < 0.001.

The nuclei were stained with Hoechst 33342 and F-actin with Alexa-fluor 568 phalloidin-conjugate and it was notably the presence of multicellular networks. The presence of many cells with elongated morphology demonstrated a good adhesion of the hMSCs to the material and not cytotoxicity. Moreover, hMSCs were embedded into the REPS-Hy during the photo-polymerization, thus producing a 3D-stem cell culture system. The scheme of the 3D-stem cell culture system preparation is shown in Figure 8A. The cell viability was evaluated after zero and three days of growth (Figure 8B,C) and 82.43 ± 0.01% cell proliferation was observed. 

These preliminary results confirm the cytocompatibility of the REPS-Hy and open the way to produce new 3D-hybrid scaffolds for tissue engineering, where the physiological role of the EPS to protect the cell from unfavorable stress and to increase the cell–cell interaction [75] could be exploited for improving the tissue formation. 

Moreover, an emerging technological and biomedical field is represented by 3D-bioprinting [76,77] of tissues and organs using stem cells and biomaterials. Photo-polymerizable hydrogels represent optimal biomaterials for this new technology for generating injectable cell/drug carrier systems.

The preparation of an appropriate bio-ink represents in 3D-bioprinting one of the main challenges. The printable biomaterials should have optimal structural and mechanical properties to drive the fate of the stem cell, while also protecting the cells from damage during printing. The peculiar intrinsic characteristics of the EPS such as the high water-uptake, dehydration resistance and the radical scavenging could be relevant properties of a bio-ink in reduce the cellular damage during the bio-printing process. Indeed, EPS can naturally inhibit the desiccation stress of the cyanobacteria, reducing the damage to cell membranes, nucleic acids and proteins induced by reactive oxygen species (ROS) under high light and UV irradiation [78]. Accordingly, with the radical scavenging property of the cyanobacterial EPS, the REPS presence in the hydrogel could reduce oxidative damages due to photo-polymerization process for 3D-bioprinting technologies. 

## 3. Materials and Methods

### 3.1. Trichormus Variabilis Biomass Cultivation

A strain of the heterocytous cyanobacterium *T. variabilis* (Kützing ex Bornet & Flahault) Komárek & Anagnostidis (VRUC168) was isolated from microphytobenthos of a Mediterranean shallow coastal lagoon (Cabras lagoon, Sardinia, Italy) [11] and maintained in standard Blue Green Medium (BG11) [79] at 18–20 °C, irradiance of 30 μmol photons m^−2^ s ^−1^ and 12:12 hs L/D cycle. A polyphasic approach was used for strain circumscription elsewhere [12]. The stock culture was acclimated at higher irradiance and temperature conditions (80 µmol photons m^−2^ s^−1^, 25 °C) and used as inoculum for biomass production. Three culture replicates were set up in 400 mL flasks in batch and the growth curves recorded by measuring the optical density (OD) at 730 nm (BECKMAN DU-65 spectrophotometer, BECKMAN COULTER, High Wycombe, UK) and the dry weight (T = 60 °C) at 24 h intervals. At the stationary phase, Day 35, each culture was used as inoculum to mass cultivate *T. variabilis* in three low-cost polyethylene bags (PBRs), of 10 L each. BG11 medium was supplied only at the beginning of the growth experiments. The PBRs were kept, at the optimized growth conditions with air mixing, in a growth cabinet equipped with white fluorescent lamps (OSRAM L 30w/956, Munich, Germany) and thermostated (25 °C). Subsamples (1 mL for OD and 5 mL for dry weight) of each PBR cultures were taken in triplicate every 48 h and the growth estimated as above. The daily biomass productivity was calculated by dividing the difference between the dry weights estimated at the end and at the beginning of the experiments by the experiment duration (days) [80]. Biomass separation was carried out at the stationary phase (Day 20) when air mixing was stopped and biomass settled at the bottom of the PBRs and the total biomass yield was evaluated after centrifuging (5000× *g* for 20 min; Heraeus SEPATECH, Megafuge 1.0, Thermo Fisher Scientific, Waltham, MA, USA) and freeze-drying.

Light microscopy observations of culture samples at the stationary phase were conducted after staining for 10 min using Alcian Blue (AB) 1%, in HCl 0.5 N (pH 0.5), specific for sulfated polysaccharidic residues present in the REPS or in 3% acetic acid (pH 2.5) specific for carboxylic moieties. A light microscope (ZEISS Axioskop, CARL ZEISS, Jena, Germany) equipped with differential interference was used and the micrographs were acquired using a digital camera Coolpix995 (Nikon, Tokyo, Japan).

### 3.2. Extraction of REPS

Released-EPS fraction was obtained according to Ahmed and colleagues procedure [81]. The culture solutions from the PBRs were centrifuged (5000× *g* for 20 min) and the supernatants containing REPS were separated from pellets. The following steps were designed and adapted to the scaling up of REPS production. Thus, the supernatants were evaporated and the REPS concentrated using a Rotavapor Buchi WaterBath B-480 at 45 °C. The concentrated supernatant was precipitated in 96% cold ethanol and the REPS fraction was obtained. The fraction was dialyzed against bi-distilled water at 4 °C for 2–3 days using a dialysis membrane with a cut-off of 18 kDa (Spectrum Laboratories, Inc., Breda, Netherlands), and then freeze-dried and stored at −20 °C for further analysis.

### 3.3. REPS Characterization

Ten milligrams of freeze-dried REPS were dissolved in 1 mL MilliQ water and total sugar content determined by the phenol-sulfuric acid method, using glucose as standard [82] (Figure S3). Total protein concentration was also assessed using BCA colorimetric assay (Sigma-Aldrich, Milan, Italy) and bovine albumin (BSA) as standard protein for calibration curve.

The RP-HPLC analysis of the REPS solutions was performed using LC-10AVP equipment (Shimadzu, Milan, Italy), 0.1% *v*/*v* trifluoroacetic acid as solvent A and 80% *v*/*v* acetonitrile and 0.1% *v*/*v* trifluoroacetic acid as solvent B. The analyses were performed using a C_18_ column (CPS Analitica, 150 mm × 4.6 mm, 5 μm), a loop of 20 μL, flow of 0.8 mL/min and the following solvent B gradient: 0–5 min, 0%; 5–50 min, 60%; 50–55 min, 60%; 55–60 min, 90%; and 60–65 min, 90%. The elution was monitored at 220 nm by a UV detector (Shimadzu, Milan, Italy).

FT-IR Spectroscopy was used for chemical analysis of freeze-dried REPS material, using a Perkin Elmer Spectrum 100 (PerkinElmer Inc., Paris, France). Spectra were acquired in the range 4000–250 cm^−1^, by averaging 32 scans at a resolution of 4 cm^−1^, using CsI cells. Data were processed using Spectrum 6.3.5 software (PerkinElmer Inc., Paris, France).

### 3.4. FAME Extraction and Characterization

FAME extraction from residual biomass and in situ trans-esterification, were carried out as previously described [13,83]. Briefly, the biomass after EPS extraction, was resuspended in a methanol and sulfuric acid (*v*/*v* 15:1) mixture for 6 h at 60 °C. After filtration, hexane was used to separate the FAME fraction. FAMEs content was estimated as the percentage of esterified lipids per dry biomass (grams). The FAME profile was determined using a gas chromatograph-mass spectrometry (Gas chromatograph GC-2010 Plus mass spectrometer GCMS-QP2010 Ultra; Shimadzu Corp., Kyoto, Japan). Eight microliters of each sample were injected into the column (SLB-5ms Fused Silica Capillary Column; 30 m × 0.25 mm × 0.25 μm film thickness) with a temperature program starting from 170 °C, increasing of 3 °C/min to 240 °C final, hold for 20 min. Split ratio was 1:80 and injection temperature 280 °C, helium was used as carrier gas. The run time for every single sample was 35 min. The identification of FA was performed by comparing the obtained mass spectra with NIST Mass Spectral Data Base (http://webbook.nist.gov/chemistry/). Total lipid concentration refers to the sum of total FAMEs.

### 3.5. REPS-Hydrogel Preparation

PEGDa 6 kDa MW (Sigma-Aldrich, Milan, Italy) and REPS solution were used for synthesizing highly cross-linked hydrogels (REPS-Hy). REPS (8.83 mg/mL), 10 mM CaCl_2_ and 2 or 3% (*w*/*v*) of PEGDa were solubilized in PBS, pH 7.4. The free-radical photo-polymerization of the hydrogels was achieved by addition of 0.1% (*w*/*v*) Irgacure^®^2959 (Ciba Specialty Chemicals, Basel, Switzerland) to the precursor solution, followed by 5 min exposure to long-wave UV light (365 nm, 4–5 mW/cm^2^). Finally, REPS-Hys were washed with sterile PBS solution to remove non-polymerized material. 

### 3.6. REPS-Hy Characterization

#### 3.6.1. Rheological Analyses of REPS-Hy

The mechanical properties of the REPS-Hy were measured using oscillatory, strain-rate controlled rheometry. The shear storage modulus (G’) of the hydrogels was determined by applying oscillatory strain and measuring the shear response, using a TA Instruments AR-G2 rheometer (ARES, TA Instruments, New Castle, DE, USA) equipped with a 20-mm parallel-plate geometry adapted with an ultraviolet (UV) light-curing assembly. The sample (200 μL) of the liquid hydrogel precursor was pipetted onto the transparent lower plate, and the upper plate was lowered until it reached a gap of ~600 μm. For hydrogel curing, the precursor was exposed from underneath the geometry to long-wave UV light (365 nm, 5 mW/cm^2^). Dynamic time sweeps were performed at 25 °C, 2% sinusoidal strain, and 3 rad/s constant frequency, while continuously monitoring the shear response of the materials before, during and after light-activated polymerization (in situ rheometry). The measurements were carried out for one minute without UV, followed by UV light activation until after the maximum value of G’ was reached (approximately 5 min). Frequency sweep measurements were performed on the hydrogels, whereby constant 2% strain at an oscillation frequency of 0.1–100 rad/s was applied while measuring the shear response. Strain sweep measurements were performed on the hydrogels whereby the oscillation frequency was held constant at 3 rad/s and strain was varied from 1% to 100%, while measuring the shear response. The sample rheology measurements were performed on two replicates for each treatment. 

#### 3.6.2. Swelling Analysis of REPS-Hy

Swelling behavior of REPS-Hydrogel was investigated over 24 h, at room temperature, until equilibrium swelling as described elsewhere [84,85]. Briefly, three replicas of REPS-Hy and PEGDa-Hy (used as control) were weighted immediately after photo-polymerization and placed into a well with 500 µL of PBS and weighed at different times: 1, 2, 6, 24 and 30 h. The degree of swelling, corresponding to the percentage of water uptake (WU), was calculated following Equation (1):

%WU = (W_t_ − W_0_)/W_0_ × 100 (1)

where W_t_ is the mass of the swollen hydrogel at time t, and W_0_ is the mass after gel-polymerization.

### 3.7. Detoxification Enzyme Synthesis and Encapsulation into the REPS-Hy

Recombinant thiosulfate:cyanide sulfurtransferase (TST, rhodanese EC.2.8.1.1) from *Azotobacter vinelandii* was produced and purified as previously described [86,87]. TST activity of the enzyme was tested using the Sörbo assay [88] obtaining an enzymatic activity of 64.06 U/mg. Briefly, the Sörbo assay was performed as follow: the recombinant TST enzyme was incubated at 37 °C in a reaction mixture (650 μL of 58 mM KCN and 58 mM sodium thiosulfate in 50 mM Tris-HCl buffer, pH 8.0). The reaction was stopped after 1 min by adding 100 µL of 15% formaldehyde and addition of 250 µL Sörbo reagent (100 g of ferric nitrate and 200 mL of 65% nitric acid per 1500 mL). The product was monitored reading the absorbance at 460 nm. The Sörbo assay for evaluating the TST activity in the presence and in the absence of REPS solution (8 mg/mL REPS, 8 mM CaCl_2_ in PBS) was performed at 23 °C after 1 min of incubation. Then, 10.08 μM of TST in 60 μL of REPS-Hy precursor solution (solution with 6.67 mg/mL REPS, 3% *w*/*v* PEGDa, 1.6% *v*/*v* Irgacure^®^2959 and PBS) was photo-polymerized for 5 min under UV light at 365 nm. The solution was photo-polymerized into a teflon mold (50 mm inner diameter) and the TST activity of the TSTREPS-Hy was assessed at different times of incubation (1, 5, 15, 30 and 60 min) of the gel at 37 °C with 58 mM KCN and 58 mM sodium thiosulfate in 50 mM Tris-HCl buffer, pH 8.0, the reaction was stopped by addition of formaldehyde and Sörbo reagent and the absorbance evaluated at 460 nm. 

### 3.8. Stem Cell Viability in 2D and 3D Cell Growth Systems

Human cardiac resident Mesenchymal Stem Cells (hMSC) line expressing stem cell marker Sca^-^1^+^ Lin^−^, was obtained as previously described [37,89]. Briefly, the cell line was obtained from cells isolated from human auricular biopsies made during the course of coronary artery bypass surgery of patients undergoing cardiac surgery after signing a written consent form for the research study, according to a joint protocol approved by the Ethic Committees of Ospedale Maggiore della Carita, Novara and University Hospital Le Molinette, Turin 2011. The cells were cultured in Dulbecco’s modified Eagle medium (DMEM) (Gibco, Monza, Italy) supplemented with 10% of fetal bovine serum (FBS) (*v*/*v*) (Gibco, Monza, Italy), 2 mM L-Glutamine (Sigma-Aldrich, Milan, Italy), 100 U/mL penicillin and 100 μg/mL streptomycin (Sigma-Aldrich, Milan, Italy) (hereafter referred to as “complete medium”) at 37 °C and with 5% CO_2_. After trypsinization, the cells were seeded onto the surface of the polymerized REPS-Hy (at a density of 1 × 10^4^ cells/cm^2^), or embedded into the hydrogels by re-suspending them in the REPS-Hy precursor solution prior the photo-polymerization procedure (at a density of 0.417 × 10^6^ cells/mL). The cell-seeded hydrogels and the cell-embedded ones were cultivated in 1 mL of complete medium in 24-multiwell plates for 14 and 3 days, respectively. The cell viability was quantified by WST-1 colorimetric assay [90]. Briefly, WST-1 assay (4-[3-(4-lodophenyl)-2-(4-nitrophenyl)-2*H*-5-tetrazolio]-1,3-benzene disulfonate) (Roche Diagnostics, Sigma Aldrich, Milan, Italy) was performed by incubating the hydrogel samples for 3 h in complete DMEM (without phenol-red) in the presence of 5% (*v*/*v*) cell proliferation Reagent WST-1 at 37 °C and in 5% CO_2_. The absorbance of the medium was evaluated using iMark™ Microplate Reader (Bio-Rad, Milan, Italy) at a 450 nm wavelength.

### 3.9. Immunofluorescence Microscopy Analyses

hMSC phenotype of the cells seeded and cultured on REPS-Hy was analyzed by immunofluorescence microscopy. Gels were washed in PBS, fixed in 4% paraformaldehyde (PFA) (Sigma-Aldrich, Milan, Italy) in PBS for 30 min at room temperature. After that, the cells were permeabilized with 0.3% Triton X-100 (Sigma-Aldrich, Milan, Italy) for 5 min and maintained in a blocking buffer (10% *v*/*v* FBS, 0.1% *v*/*v* Triton X-100 and 1% *w*/*v* glycine in PBS) overnight at 4 °C. Hydrogels were incubated with F-actin 488-Alexa fluorochrome-conjugated phalloidin (Life Technologies, Milan, Italy). Nuclei were stained with 1:25,000 *w*/*v* Hoechst 33342 (Sigma-Aldrich, Milan, Italy) in PBS. Hydrogels were stored at 4 °C in 20 mM Gly PBS under dark conditions. Confocal microscopy was performed using Nikon Eclipse Ti (Nikon, Tokyo, Japan) and the signal was detected using EZ C.1 software (Nikon, Tokyo, Japan).

### 3.10. Statistical Analysis

GraphPad Prism version 6.0 program (GraphPad Software, San Diego, CA, USA) was used for statistical analysis. Three or five independent experiments were performed for each result and the analysis of the variables was made using ANOVA One-way test or the one-tailed Student’s *t*-test. A *p*-value of <0.05 was considered to be statistically significant. Standard deviations (SD) or the standard errors (SEM) of the mean were calculated and reported for each sample.

## 4. Conclusions

In this work, efficient biomass production by a native brackish strain of the cyanobacterium *T. variabilis* was obtained in a low-cost PBR to optimize a sequential extraction protocol for the EPS and PUFA production. This strain showed suitable productivity in the 10 L bags used, after 20 days without nutrient repletion, allowing us to collect sufficient biomass to be exploited for the target application. The potential to obtain an integrated and economically advantageous production of nutraceuticals and biomaterials was demonstrated. REPS fraction was characterized and used for producing a new photo-polymerizable hybrid-hydrogel. REPS-Hy was characterized for its physical, mechanical and biological properties and for its ability to embed detoxification enzymes with catalytic cysteine residues, such as TST. The results herein presented show the possibility to fabricate new functional non-cytotoxic hydrogels with enzymatic activity for therapeutic and environmental applications. The presence of carboxyl groups in the REPS could also help to produce hydrogels that exhibit pH-sensitive swelling behavior that could increase their pore size at basic pHs and allow the protein release from the matrix in the intestinal environment, significantly improving the absorption of the protein-drugs, as already demonstrated for insulin embedding hydrogels [91,92]. Moreover, in a proof-of-concept experiment, REPS-Hy was also used for fabricating 3D-cell embedding hydrogels, demonstrating the feasibility for the production of cell-carrier systems in cell therapy and of photo-printable bio-inks for tissue engineering applications. This is a first and preliminary study, which combines the multiple inexpensive production of cyanobacteria nutraceutical products with that of photo-polymerizable hydrogels for enzyme- and stem cell-carrier systems. Although the data reported suggest a good potential feasibility, further experiments are necessary to demonstrate the in vivo applicability.

## Figures and Tables

**Table 1:** Fatty acid methyl esters pattern (FAME % *w*/*w*) obtained from *T. variabilis* biomass.

Fatty Acids			% FAME *w/w*		
Systematic name	Common name	Number of carbon atoms:double bond(s)	Family	Mean *	SEM ^1^
Decanoic acid	Capric acid	C10:0	Saturated	0.61	0.03
Tetradecanoic acid	Myristic acid	C14:0	Saturated	0.70	0.01
Hexadecenoic acid	Palmitoleic acid	C16:1 (n-7)	Monounsaturated	15.25	1.34
Hexadecadienoic acid		C16:2 (n-4)	Polyunsaturated	5.28	0.54
Octadecanoic acid	Stearic acid	C18:0	Saturated	0.83	0.19
Octadecenoic acid	Oleic acid	C18:1 (n-7)	Monounsaturated	3.05	1.36
Octadecadienoic acid	Linoleic acid	C18:2 (n-6)	Polyunsaturated	24.46	1.91
Octadecatrienoic acid	α-Linolenic acid	C18:3 (n-3)	Polyunsaturated	27.71	2.33
Eicosanoic acid	Arachidic acid	C20:0	Saturated	22.11	4.60
SAFA				24.25	4.76
MUFA				18.29	0.02
PUFA				57.45	4.77

^1^ SEM refers to standard error; * Mean of three replicates.

## Supplementary Materials

The following are available online at http://www.mdpi.com/1660-3397/16/9/298/s1, Figure S1: REPS production over time; Figure S2: RP-HPLC analysis of the TST solution and of the fraction released from the REPS-Hy; Figure S3: Biochemical analysis of REPS.

## Abbreviations

3D	three-dimensional
AB	Alcian Blue
BG11	Blue-Green Medium
BCA	bicinchoninic acid
BSA	Bovine serum albumin
BSCs	Biological Soil Crusts
hMSCs	human Lin^-^Sca-1^+^ cardiac mesenchymal stem cells
DMEM	Dulbecco’s Modified Eagle Medium
EPS	Extracellular Polymeric Substances
ePBR	emerse photobioreactor
FA	fatty acids
FAME	Fatty Acid Methyl Esters
FBS	fetal bovine serum
FT-IR	Fourier Transform Infrared Spectroscopy
Hy	hydrogel
MUFA	monounsaturated FA
PBR	photobioreactor
PUFA	polyunsaturated FA
PEGDa	PEG-diacrylate
PFA	paraformaldehyde
REPS	Released Extracellular Polymeric Substances
REPS-Hy	REPS PEGDa-hydrogel
RP-HPLC	Reversed Phase-High-Performance Liquid Chromatography
SAFA	saturated FA
SD	standard deviations
SEM	standard errors of the mean
TST	thiosulfate: cyanide sulfur transferase
TFA	trifluoroacetic acid
WST-1	4-[3-(4-lodophenyl)-2-(4-nitrophenyl)-2*H*-5-tetrazolio]-1,3-benzene disulfonate
WU	water uptake
