# Supplementary material for: Trichormus variabilis (Cyanobacteria) Biomass: From the Nutraceutical Products to Novel EPS-Cell/Protein Carrier Systems
Source: Mar Drugs. 2018 Aug 27;16(9):298. doi: 10.3390/md16090298 (PMC6164293; doi:10.3390/md16090298)
Supplement: Supplementary file 1 [file marinedrugs-16-00298-s001.pdf]

Supplementary Materials

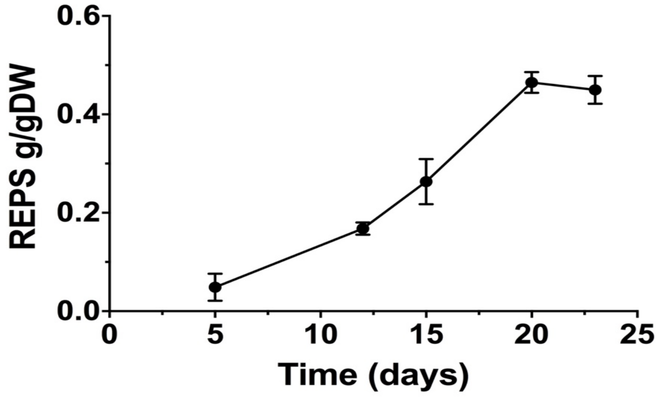

Figure S1. REPS production over growth. REPS concentration over one 23 day growth experiment, at about 5 day intervals. REPS concentration data: day 5 =48 mg/gDW; day 12=168 mg/gDW; day 15=263 mg/gDW; day 20= 465 mg/gDW, day 23=450 mg/gDW.

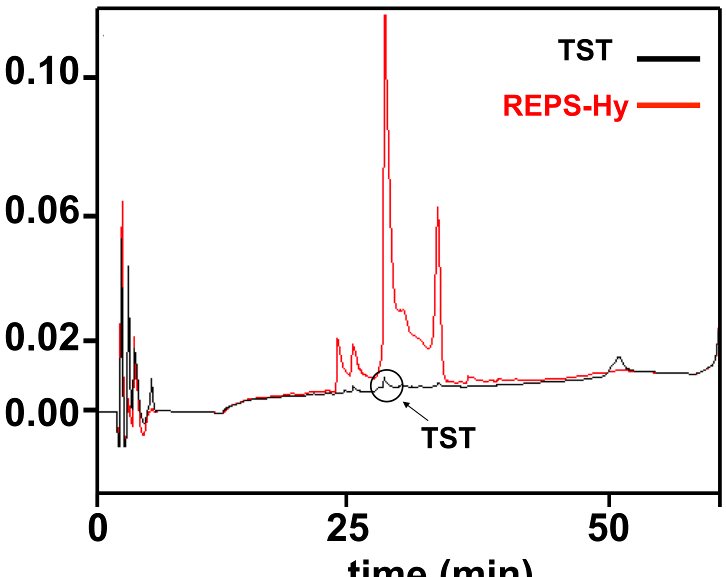

Figure S2. RP-HPLC analysis of the fraction released from the REPS-Hy and of TST solution. Overlapping of the RP-HPLC profiles of 100 µl of 10.08 µM of TST (—) and of the soluble fraction released from REPS-Hy after 20h of incubation at 37°C (—) in 200 µl of 50 mM Tris-HCl buffer, pH 8.0. Solvent A was 0.1% v/v TFA and solvent B 80% v/v acetonitrile and 0.1% v/v TFA. The analyses were performed using a C_{18} column (CPS Analitica, 150 mm× 4.6 mm, 5 µm) at 0.8 ml/min flow rate and the following solvent B gradient: 0–5 min 0%; 5–50 min 60%; 50–55 min 60%; 55–60 min 90% and 60–65 min 90%. The elution was monitored at 220 nm.

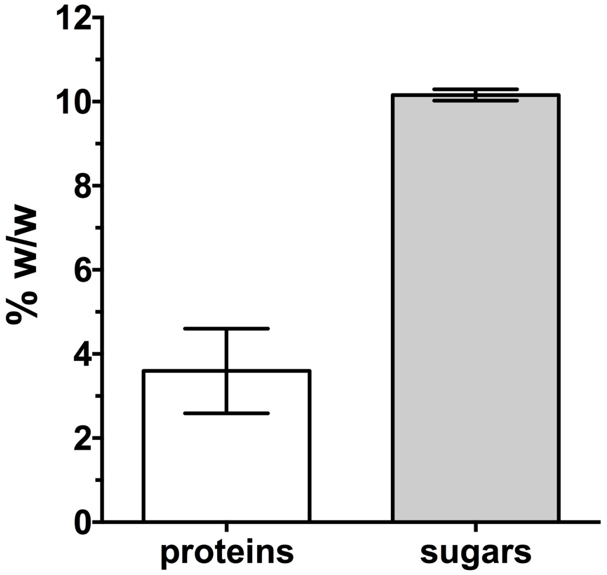

Figure S3. Biochemical analysis of REPS. Percentage of proteins and sugars present in REPS obtained by BCA and Dubois assays respectively.
